# Chemical–Physical Characterization of PET-G-Based Material for Orthodontic Use: Preliminary Evaluation of micro-Raman Analysis

**DOI:** 10.1055/s-0043-1764424

**Published:** 2023-05-29

**Authors:** Fabiana Nicita, Cesare D'Amico, Vincenzo Filardi, Donatella Spadaro, Emidio Aquilio, Maura Mancini, Luca Fiorillo

**Affiliations:** 1 Department of Biomedical and Dental Sciences and Morphofunctional Imaging, University of Messina, via Consolare Valeria, Messina, Italy; 2 D.A. Research and Internationalization, University of Messina, Via Consolato del mare 41, Messina, Italy; 3 CNR-IPCF, Institute for Physical Processes, Messina, Italy; 4 Lineo Aligners, Torino, Italy; 5 Multidisciplinary Department of Medical-Surgical and Dental Specialties, University of Campania Luigi Vanvitelli, Naples, Italy

**Keywords:** clear aligners, PET-G, thermoplastic materials, staining substances, chemical–physical characterization, micro-Raman spectroscopy.

## Abstract

**Objectives** Orthodontic treatment with clear thermoplastic aligners is in great demand by patients especially for aesthetics. Any alterations in the chemical composition of the thermoplastic material for aligners, subjected to the oral environment and exposure to various commonly used substances, could influence the desired orthodontic movement decreasing the predictability of the treatment. The objective of this study was to determine the chemical–physical characterization by micro-Raman spectroscopy of a thermoplastic material based on polyethylene terephthalate glycol (PET-G) used for the manufacture of Lineo aligners (Micerium Lab, Avegno, Italy) subjected to different staining beverages and cleaning agents.

**Materials and Methods** Twenty-two thermoformed PET-G samples were immersed to various substances of daily use for 10 and 15 days (coffee, tea, Coca-Cola, red wine, colloidal silver disinfectant, nicotine, artificial saliva, cigarette smoke, and different combinations of saliva with some of the previous solutions). Subsequently, the chemical–physical characterization was investigated by micro-Raman spectroscopy.

**Results** The analysis of the spectra acquired for all the specimens showed no difference in the exposure to the different solvents at 10 and 15 days. Furthermore, having ascertained the heterogeneous surface morphology of the PET-G material due to thermoforming, various deposits were present on all the samples whose consistency and concentration depended on the substance used.

**Conclusion** The spectroscopic investigations have provided a precise and detailed analysis of the qualitative and structural data of the PET-G material under examination. No significant structural modifications of the thermoplastic polymer were found after immersion in different solutions in the exposure times adopted.

## Introduction

Current computer-aided design and manufacturing (CAD/CAM) techniques are widely used in the manufacture of clear thermoplastic aligners for the treatment of various types of dental malocclusion. 1 2 3 4 The biomechanical characteristics of commercial clear aligners are influenced by the properties of the thermoplastic material used, the forming process, 2 5 and the precise aligner-teeth fitting. 6

The thermoplastic polymers currently most used are based on polyurethanes (PU) or polyesters such as polyethylene terephthalate glycol (PET-G). 7 8 9 These materials have good mechanical and physical properties, biocompatibility, chemical stability, excellent aesthetic characteristics, good formability, and are of low cost, 10 but also have various limitations such as low strength and poor wear resistance. 11 In particular, PET-G materials are structurally amorphous polymers that possess an irregularly arranged molecular structure with poor packaging. 12 These polymers are transparent because they are passed through by visible light and their degree of crystallization after thermoforming is negligible. 13

However, the thermoforming could cause variations in the morphological and mechanical properties of the polymer. 5 Furthermore, during their use, thermoplastic aligners are continuously subjected to mechanical loads, temperature changes inside the oral cavity, and the influence of saliva and food pigments. 14 These factors could lead to abrasions, surface alterations, color changes, and delamination of the polymer. 14 15 16 Chemical cleaning methods may also alter the structural integrity of the thermoplastic material. These degradations lead to a significant loss of mechanical strength and transparency of the aligner, an essential requirement in an invisible orthodontic treatment. 13 17

Although clinicians recommend removing aligners before eating and drinking, patient compliance is still insufficient. Therefore, it is necessary to simulate the intraoral clinical conditions, even with the use of staining substances and agents such as nicotine and cigarette smoke, to better evaluate any structural and/or optical changes of the orthodontic aligners. The aim of this research is to characterize the PET-G-based thermoplastic material used for Lineo aligners (Micerium Lab) after exposure to various commonly used staining and disinfecting agents. In detail, micro-Raman spectroscopy was used to identify any changes in the chemical composition with consequences on the morphology and/or structure of the polymer in question.

## Materials and Methods

### Preparation of PET-G Samples

**Fig. 1:**
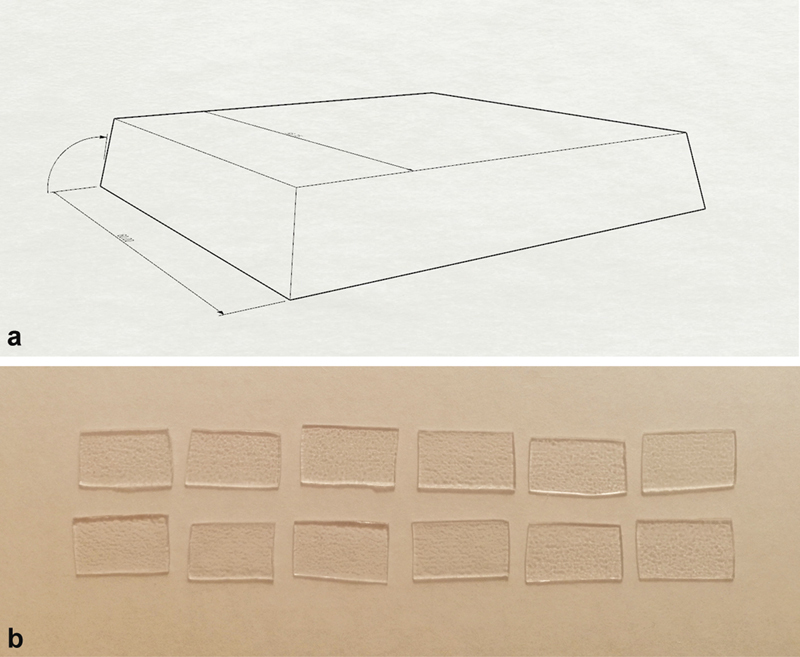
Preparation of thermoformed polyethylene terephthalate glycol (PET-G) samples. ( **a** ) Truncated pyramid model used in the thermoforming phase. ( **b** ) Some of the thermoformed samples cut from the horizontal surface of the model obtained.

Four sheets of thermoplastic material in PET-G with a thickness of 0.8 mm were selected for the study. Three of these slabs were thermoformed, while the fourth was not subjected to hot molding techniques. A truncated pyramid-shaped mold was prepared and placed in the thermoforming machine. A thermoforming quota of 20 mm and an angle of 11 degrees was applied for less friction in extraction. The applied temperature is 160 to 165°C. The models obtained after thermoforming were removed and the horizontal surface was used to obtain samples with dimensions of 1 × 1.5 cm ( *n* = 23; Fig. 1 ). A nonthermoformed sample of 1 × 1.5 cm was obtained from the fourth sheet, which will be used to evaluate the influence of thermoforming on the PET-G material.

### Treatment of PET-G Samples with Commonly Used Substances

It is essential to evaluate whether the intake of beverages or the use of certain substances or detergents used in cleaning the aligners can influence the chemical–physical characterization of the thermoplastic PET-G material under analysis. Twenty-two thermoformed samples were used for this purpose. Each specimen was placed in a plastic container containing 5 mL of one of the following solutions: (1) 6 g of coffee powder (Nescafé Classic, Switzerland) was added to 200 mL of boiling deionized water; (2) sugar-free tea (30 mg/150 mL, Earl Grey Twinings, London, England); (3) Coca-Cola (Coca-Cola Company, Atlanta, Gorgia, United States); (4) undiluted red wine (San Crispino, Cantine Ronco Romagna, Italy); (5) disinfectant based on colloidal silver; (6) the nicotine solution was obtained by infusing cigarette filters into deionized water and then filtered; (7) 120 mL of artificial saliva (Biotène Oral Balance, GlaxoSmithKline Consumer Healthcare S.p.A.) were diluted in 480 mL of deionized water; (8) cigarette smoke (a certain amount of smoke produced by cigarettes); (9) saliva and smoke; (10) solution of saliva and nicotine in a 1:1 ratio; and (11) a solution of saliva and coffee in a 1:1 ratio.

All specimens were maintained in immersion in a water bath at T = 37°C and were randomly divided according to the immersion time in the chosen solvents ( *n* = 11 up to 10 days; *n* = 11 up to 15 days). The samples were rapidly rinsed with deionized water (Milli-Q) every 24 hours before being reimmersed in fresh solution.

As for the colloidal silver-based disinfectant, the two thermoformed samples, up to 10 and 15 days, were treated for a maximum of 3 hours a day to simulate the aligner cleaning methods.

**Fig. 2:**
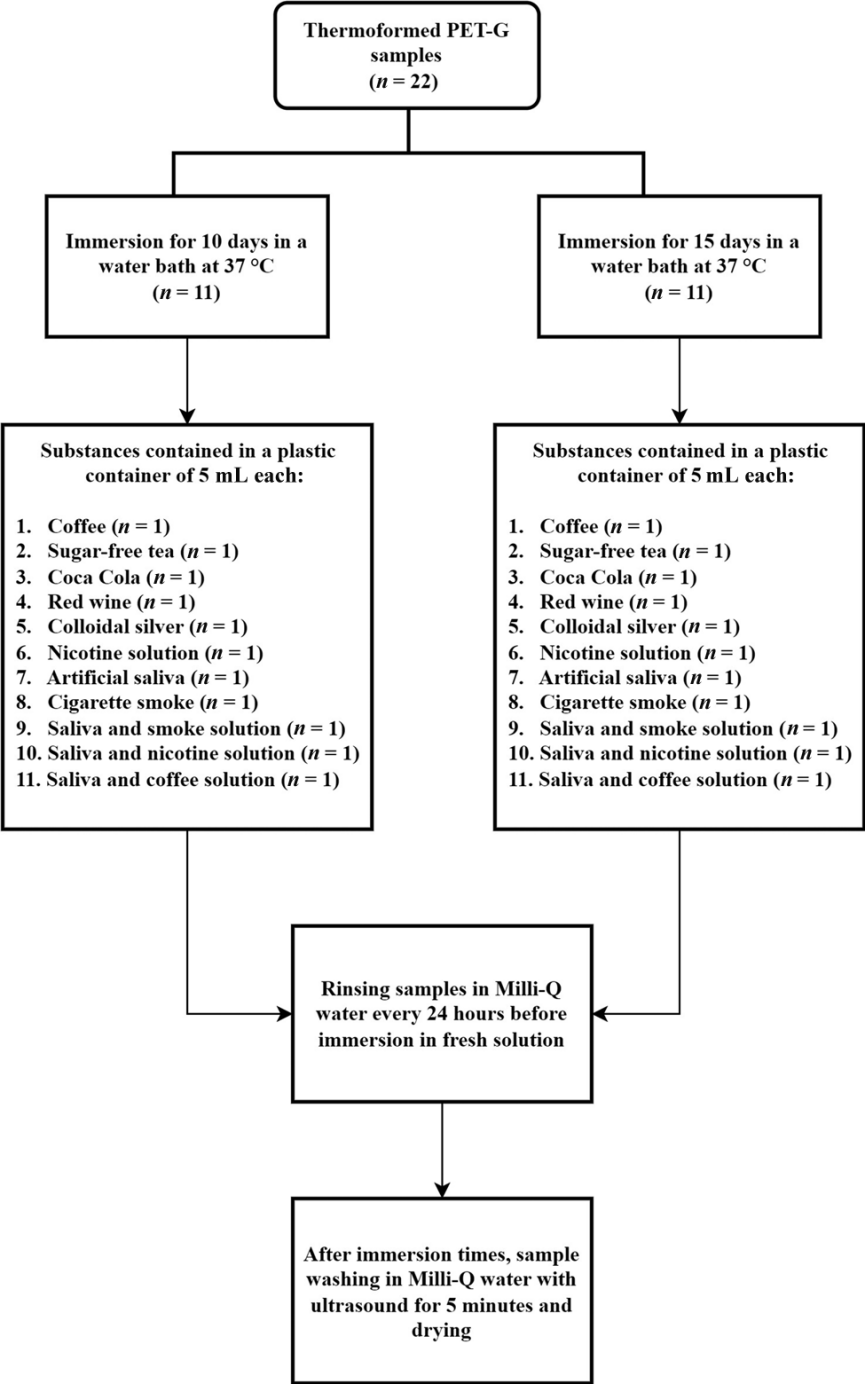
Flowchart of the protocol adopted for the treatment of polyethylene terephthalate glycol (PET-G) samples immersed in staining substances.

After 10 and 15 days, all the samples were washed by immersion in Milli-Q water, kept in ultrasound for 5 minutes and adequately dried before being analyzed for the physicochemical characterization ( Fig. 2 ).

### Micro-Raman Spectroscopy Analysis

Raman spectroscopy analysis was performed to investigate the chemical composition of the samples in PET-G. The measurements were carried out with a micro-Raman spectrometer (XploRA Plus, Horiba) using a laser source with a wavelength of 785 nm with a 100X objective, 1,200 g/mm reticle, with 10-second acquisition time for five accumulations in a range between 200 and 3,200/cm.

## Results

### Influence of the Thermoforming Process

**Fig. 3:**
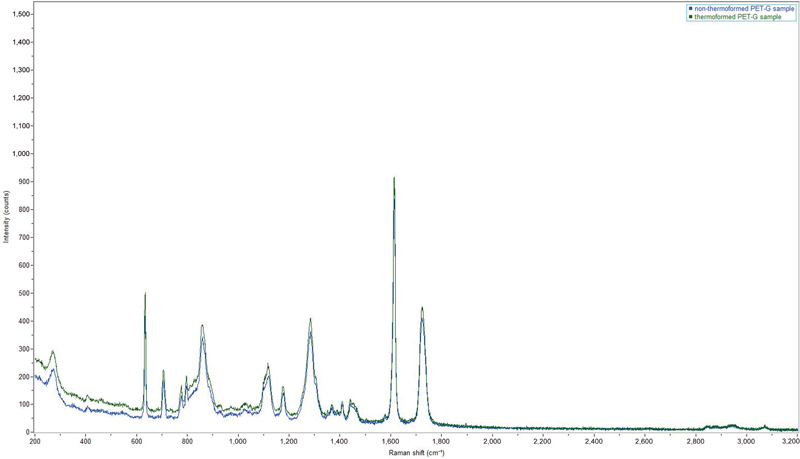
Raman spectra of non-thermoformed ( *blue* ) and thermoformed ( *green* ) polyethylene terephthalate glycol (PET-G) samples at 785 nm. The characteristic peaks belong to the PET-G polymer and there is no change in the signals when heat treatment is carried out.

**Fig. 4:**
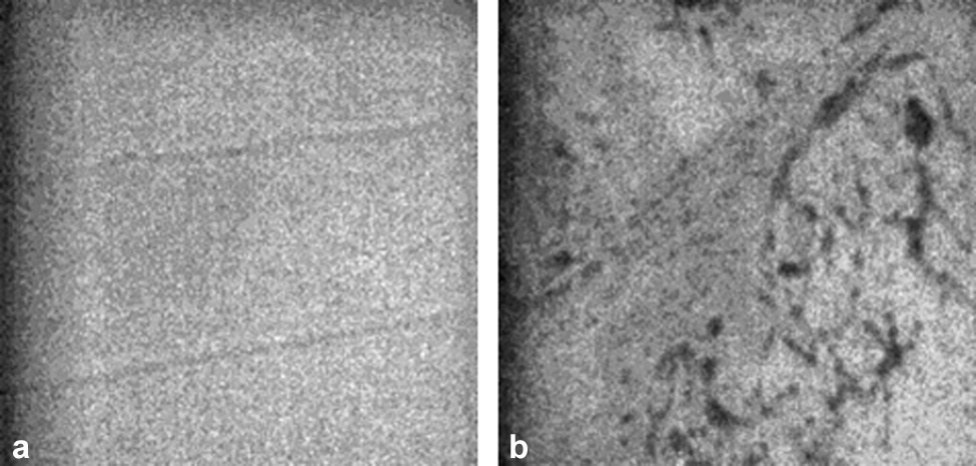
Morphological analysis under an optical microscope at 100x magnification. ( **a** ) Smooth and homogeneous non-thermoformed surface and ( **b** ) thermoformed surface with scratches and roughness.

The peaks characteristic of the vibrational modes of PET-G 18 are shown in Table 1 . To assess the influence of the thermoforming process in the physicochemical characterization, two PET-G samples were compared ( *n* = 1 thermoformed and *n* = 1 nonthermoformed). The preliminary investigation shows that the structure of the polymer used is not altered by the thermoforming process ( Fig. 3 ). However, the morphological analysis shows that the surface of the thermoformed sample is characterized by roughness, holes, and scratches in comparison with the uniform and homogeneous surface of the one not subjected to thermoforming ( Fig. 4 ).

**Table 1:** The assignment of observed micro-Raman bands of polyethylene terephthalate glycol (PET-G)

Raman (per cm)	Assignment
3,082 w	CH stretching
3,068 w	CH stretching
3,000 w	CH stretching
2,960 w	CH _2_ stretching
1,730 m	C = O stretching
1,615 s	C = C stretching (ring)
1,450 w	CH deformation
1,412 vw	C–C stretching (ring)
1,370 vw	CH _2_ wagging
1,282 m	C–C stretching (ring), C–O stretching
1,270 m	C–C stretching (ring), C–O stretching
1,245 sh, w	C–C stretching (ring), C–O stretching
1,175 w	CH in plane bending (ring)
1,117 w	CH in plane bending (ring), C–O stretching
1,038 vw	C–C stretching (glycol)
890 vw	CH _2_ rocking
853 w	C–C stretching (ring breathing), C–O stretching
797 w	CH out of plane bending (ring)
633 s	CCC in plane bending (ring)
407 vw	Asymmetric ring torsion
272 w	C–C stretching (ring), CCC bending (ring)

Abbreviations: m, moderate; s, strong; sh, shoulder; w, weak; v, very.

### Aging Treatments

**Fig. 5:**
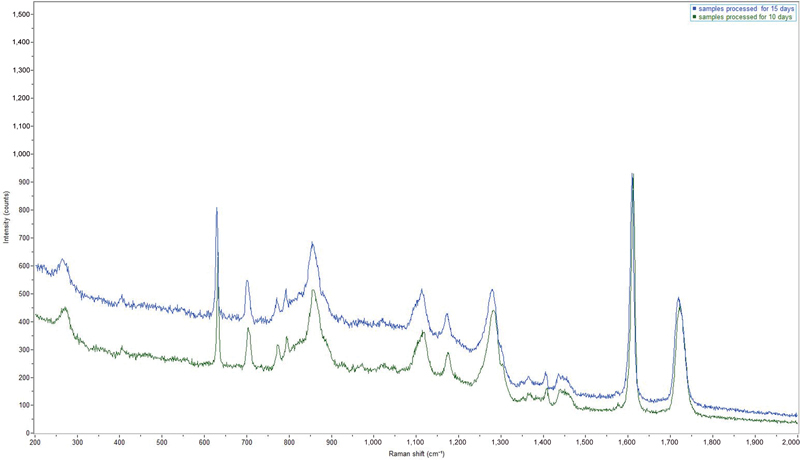
Raman spectra representative of the signals found on the surface of the samples subjected to the different treatments for 10 ( *green* ) and 15 days ( *blue* ). Peaks found are those characteristics of the polyethylene terephthalate glycol (PET-G) polymer and are stackable with each other.

From the analysis of the Raman spectra acquired for all the samples immersed in the different substances ( *n* = 22), no differences were found between those treated for 10 and 15 days ( Fig. 5 ). Therefore, the results shown are comparable to the single solvent or agent used regardless of the duration of the treatment itself.

**Fig. 6:**
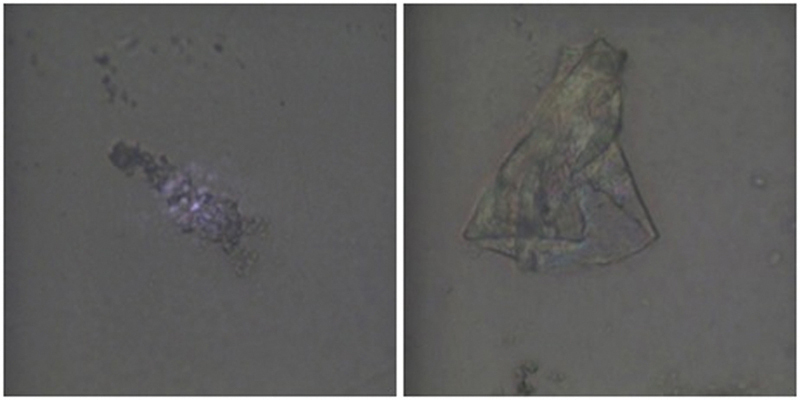
( **a,b** ) Morphological analysis of polyethylene terephthalate glycol (PET-G) samples immersed in a coffee solution. There is presence of residues with a discrete diffuse fluorescence.

The micro-Raman analysis shows that in all the samples the acquired spectra are those of the PET-G polymer, whatever the agent used in the treatments at both 10 and 15 days. Only in the samples dipped in nicotine were areas of burns and morphological changes of the polymer detected. Furthermore, in all the samples, different fluorescence phenomena are found due to residues of substances accumulated in correspondence with the asperities and scratches of the thermoformed material ( Figs. 6 7 8 9 10 11 12 ). There are also fibrous residues of accidental origin attributable to cotton fibers on the surface of the samples immersed in the saliva and coffee solution, at 10 and 15 days, and treated with colloidal silver disinfectant for 15 days ( Fig. 13 ).

**Fig. 7:**
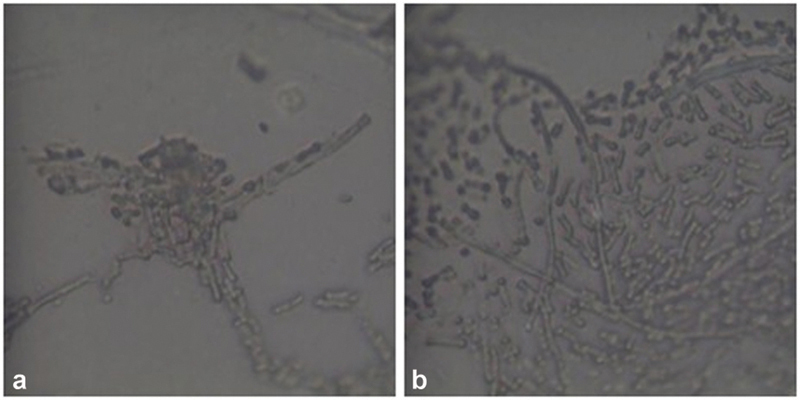
( **a,b** ) Morphological analysis of polyethylene terephthalate glycol (PET-G) samples immersed in a sugar-free tea. There is presence of highly fluorescent tree structures.

**Fig. 8:**
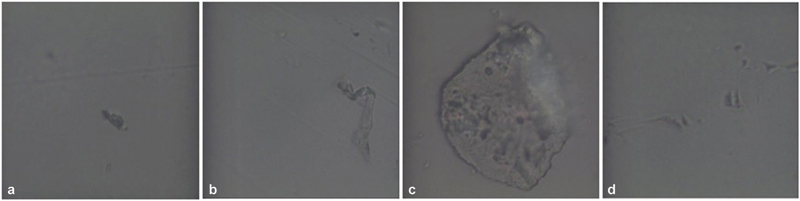
Morphological analysis of polyethylene terephthalate glycol (PET-G) samples immersed in ( **a,b** ) Coca-Cola and ( **c,d** ) red wine. There are no structures and no fluorescence.

**Fig. 9:**
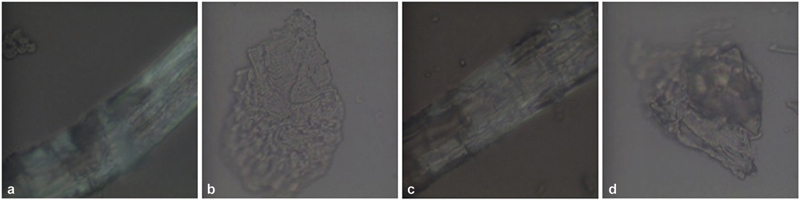
Morphological analysis of polyethylene terephthalate glycol (PET-G) samples immersed ( **a,b** ) in a silver colloidal and ( **c,d** ) in the solution of artificial saliva and coffee. Along with the usual structures, large nonfluorescent cottonlike fibrous structures are present.

**Fig. 10:**
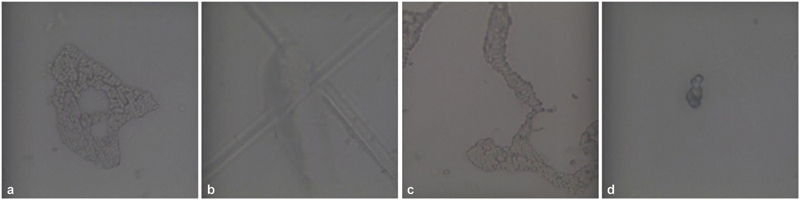
Morphological analysis of polyethylene terephthalate glycol (PET-G) samples immersed ( **a,b** ) in a pure nicotine and ( **c,d** ) in the solution of artificial saliva and nicotine. There is presence of carbon deposits and rodlike structures, which cause less fluorescence.

**Fig. 11:**
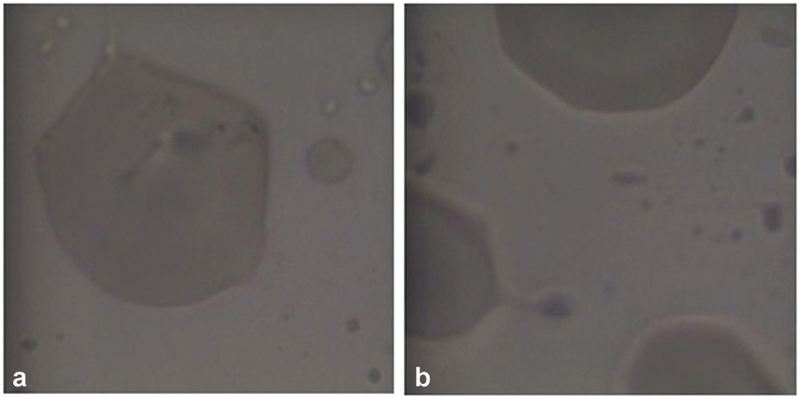
( **a,b** ) Morphological analysis of polyethylene terephthalate glycol (PET-G) samples immersed in an artificial saliva and saliva with cigarette smoke. There is presence of almost regular circular spots without fibrous or rodlike structures.

**Fig. 12:**
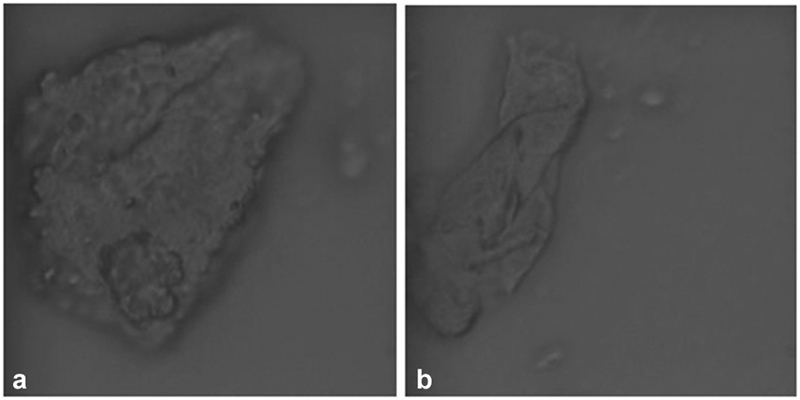
( **a,b** ) Morphological analysis of polyethylene terephthalate glycol (PET-G) samples immersed in cigarette smoke. There is presence of deposits with little fluorescence, without fibrous structures or rods or circular spots.

**Fig. 13:**
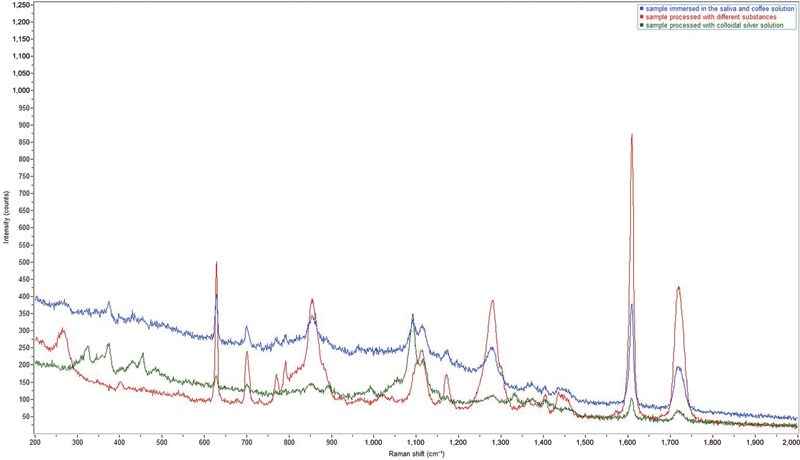
Raman spectra representative of some signal types found on the surface of the polyethylene terephthalate glycol (PET-G) samples. The observed peaks correspond to those of cotton-fiber-like foreign bodies found on the surface of samples immersed in saliva and coffee solution ( *blue* ) and in those treated with colloidal silver ( *green* ) in comparison with a representative Raman spectrum of treated samples with other substances ( *red* ).

## Discussion

The qualitative properties of clear orthodontic aligners are strictly related not only to the material used and the molding process for manufacturing but also to modifications related to the oral environment. The thermoforming process applied to thermoplastic materials involves a heating cycle followed by forming under vacuum or pressure, which could cause morphological alterations of the polymer. 5 Furthermore, these materials in the form of aligners inserted inside the oral cavity can also be subject to surface and color alterations due to factors such as humidity, temperature variations, and coloring agents. 5 13 19 Despite clinicians' recommendations to wear aligners full-time except when eating, drinking, or for oral hygiene maneuvers, 20 many patients take coloring agents (red wine, coffee, tea, Coca-Cola) or smoke with their orthodontic devices. 21 22

This *in vitro* study reports the chemical–physical characteristics of PET-G, widely used for the fabrication of orthodontic aligners (Lineo, Micerium Lab), which has undergone various aging treatments. Micro-Raman spectroscopy is a nondestructive chemical analysis technique that provides detailed information on the chemical structure, phases, and polymorphy and molecular interactions. It is integrated with an optical microscope, which, by focusing the laser on the sample, allows the analysis of small regions. 23

The originality of this work consists in the chemical–physical characterization of the thermoplastic material that occurs after its exposure to substances for common use for a prolonged time (15 days) equal to the period of use of a single aligner during complex orthodontic treatment. This has great relevance in observing the structural behavior of the polymer during intraoral aging.

Although the micro-Raman analysis did not reveal any changes in the chemical structure of PET-G ( Fig. 3 ), the thermoforming process used to manufacture the aligners caused evident surface alterations of the polymer including roughness, holes, and scratches ( Fig. 4 ). Also, Porojan et al 24 noted an increase, albeit insignificant, in the roughness of this material. In contrast, the study by Zhang et al 25 showed a smoother PET-G surface after thermoforming.

Alterations in the physical properties of PET-G have also been reported after intraoral exposure. 25 The micro-Raman analysis performed after immersion in different solvents for up to 10 and 15 days has shown that the structure of PET-G is not altered and that the fluorescence phenomena found are due only to the accumulation of residues of the substances used ( Figs. 6 7 8 9 10 11 12 ). The only morphological changes were detected in the nicotine-treated samples as the interaction between the micro-Raman laser and the remaining carbon residues resulted in local burn areas of the material. Furthermore, in the samples immersed in the solution of saliva and coffee and in those treated with colloidal silver disinfectant, accidental foreign bodies of a fibrous nature were detected, whose composition and characteristic peaks are probably related to cotton fibers ( Fig. 13 ). 26

Transparency represents another important property possessed by thermoplastic materials. 27 Any color changes have been associated with the absorption or penetration of the coloring substances that come into contact with the surface of the material within the oral environment. 17 28 29 The surface characteristics of the thermoformed materials could accelerate the accumulation of pigment with consequent loss of transparency. 30 Daniele et al 9 found alterations in the aesthetic characteristics of aligners made of PET-G and PU, especially after immersion in coffee or red wine, with a consequent loss of transparency due to impurities deposited on the surface. However, Porojan et al 24 have shown that color variations of the PET-G samples do not depend so much on the surface roughness but on the penetration inside the material itself.

In the present study, no substantial variations in the colorimetric level were found; however, the spectroscopic technique used in this study does not have the ability to detect any changes of this type. Therefore, in future research, it would be appropriate to evaluate transparency changes and colorimetric variations of PET-G with the length of time used in this study (10 and 15 days) with more suitable instruments.

## Conclusions

After thermoforming and aging treatments, there are no structural or color variations on the PET-G material used for the manufacture of Lineo aligners produced by Micerium Lab.

The heterogeneous morphological surface, which could suggest a loss of transparency and aesthetic properties, depends on the intraoral exposure and on the contact with coloring pigments deriving from beverages, nicotine, or cigarette smoke, which determine the accumulation of deposits in the rough surface and scratches due to thermoforming. Therefore, the surface characteristics of the PET-G samples found on the scanning electron microscope (SEM) images after 10 and 15 days of immersion times are attributed exclusively to the staining substances used.
